# Taxonomy of new relatives of *Onthophagus catenatus* Lansberge, 1883 from New Guinea (Coleoptera, Scarabaeidae, Scarabaeinae)

**DOI:** 10.3897/zookeys.251.3994

**Published:** 2012-12-18

**Authors:** Jan Krikken, Johannes Huijbregts

**Affiliations:** 1 NCB Naturalis, PO Box 9517, NL-2300 RA, Leiden, The Netherlands; 2 NCB Naturalis, PO Box 9517, NL-2300 RA, Leiden, The Netherlands

**Keywords:** *Onthophagus*, New Guinea, *catenatus* group, key, list of species, new species

## Abstract

Four new taxa from New Guinea are proposed in the dung beetle genus *Onthophagus* Latreille, 1802, all in the operational group of *Onthophagus catenatus* Lansberge, 1883. The group is discussed, defined, and the five taxa included are listed, keyed, and diagnosed. Three new species are described: *Onthophagus abmisibilus* (from West New Guinea, Indonesia), *Onthophagus kokodanus*, *Onthophagus kokosquamatus* (both from Papua New Guinea). One new species comprises a lowland and an upland subspecies: *Onthophagus kokodanus kokodanus* and *kokodanus hagenaltus* (both in Papua New Guinea).

## Introduction

The onthophagine dung beetle fauna of New Guinea and nearby islands has been the subject of various recent papers, including a general discussion with descriptions of a diverse array of new species (Krikken and Huijbregts 2012), and a separate treatment of small, unicolour new species (Krikken and Huijbregts in press), all in the subcosmopolitan mega-genus *Onthophagus* Latreille, 1802. The present paper deals with four new species-group taxa similar to the New Guinea *Onthophagus catenatus* Lansberge, 1883 in features of their vertexal armature, size range, and various other characters. Major males in this operational group have a vertexal pair of long, evenly curved, laterally directed horns, lacking accessory inward lobes and denticles; they differ from the members of the closely related *Onthophagus tauroides* Gillet, 1930, group (cf. Krikken and Huijbregts 2012) by the horns at their base being distinctly contiguous, showing a low, variably shaped but relatively simple interconnecting elevation. The pronotum may be slightly modified (depressed, bluntly bifid, etc.), but never with deep concavities and/or strong, sharp protrusions. Note that the material available is limited, particularly from a geographic point of view, and therefore this paper should, after the last overview of Balthasar (1969), be considered just another partial update, attempting to make more taxonomic sense of the morphological diversity now seen in this set of *Onthophagus* species.

This *Onthophagus catenatus* group as here conceived is indeed diverse, and the group concept may even be insufficiently inclusive, as will be clear from the comments below. Formal group delimitation and species identification are, as usual among Scarabaeinae, complicated by intraspecific polymorphism – males, for instance, may look like females, and some species (here excluded) may never have males with long, curved, laterally directed horns. One form (*Onthophagus kokodanus kokodanus* subsp. n.) has an isolated conical median tubercle just in front of the base of its broad male vertexal horns, possibly the result of a morphoclinal forward-shifting of the medially angulate interconnecting ridge seen in other group members. Another species (*Onthophagus kokosquamatus* sp. n.) has a peculiarly rugulate-squamate pronotal surface and unusual lateral pronotal margins. This species stands apart from the others, and may be related to two much smaller named species here excluded from the *catenatus* group (see Comments under *Onthophagus kokosquamatus*). Both sexes of *Onthophagus kokosquamatus* have a similar, usually well-developed vertexal armature, contrary to at least two of the other species, in which the vertexal armature of females is reduced to a simple or more complex transverse ridge, its shape apparently different according to species.

The key to the species given below takes all this into account, the first couplet firmly delimiting the *catenatus* group from other Papuasian *Onthophagus* with long horns, in spite of the afore-mentioned complications. *Onthophagus catenatus* Lansberge, 1883, is apparently a widespread species, the label data we have seen indicate a range of altitudes from the Aru Islands to the Mount Hagen highlands and beyond. Although there is some variation, certainly needing further study, our present concept of the species (cf. also Comment in species account) is consistent with van Lansberge’s (1883) description, which mentioned the characteristic strengthening of the punctures in the elytral striae, from base to apex.

All four species included in the *Onthophagus catenatus* group are formally placed in the nominotypical subgenus.

## Material and methods

The material used in this work mainly comes from the Canadian Museum of Nature (Ottawa), and comprises 5 species-group taxa, 22 collection records, 166 specimens. The data given in the lists closely follow the information on the specimen labels. Paratypes, where available in sufficient numbers, will in due course be distributed to other collections.

Body part measurements in the descriptions were taken through the microscope with a calibrated ocular scale, and rounded off to 0.1 mm; approximate total body lengths (given for specimens as is) rounded off to ½ mm. For more background, references, and terminological and technical matters, see our companion papers mentioned above. One terminological addition: hemi*-*punctures are those that have about half of their peripheral rim effaced, usually having a seta more or less decumbent to the effaced side.

### Collection abbreviations

CMNCCanadian Museum of Nature, Ottawa, Canada


IRSNBInstitut Royal des Sciences Naturelles de Belgique, Brussels, Belgium


RMNHNational Museum of Natural History / NCB Naturalis, Leiden, The Netherlands


### List of *Onthophagus* taxa treated in this paper

*Onthophagus abmisibilus* sp. n. – Indonesia: West New Guinea (Star Mountains)

*Onthophagus catenatus* Lansberge, 1883 – Papua New Guinea, Indonesia: West New Guinea

*Onthophagus kokodanus* sp. n. – Papua New Guinea

*Onthophagus kokodanus kokodanus* subsp. n. – Papua New Guinea (E of Port Moresby)

*Onthophagus kokodanus hagenaltus* subsp. n. – Papua New Guinea (Western Highlands)

*Onthophagus kokosquamatus* sp. n. – Papua New Guinea (E of Port Moresby)

### Key to species and subspecies (males)

**Table:** 

1	Vertex of major males with pair of laterally divergent, long, evenly, upward curved horns, in various ways connected at base (usually by transverse elevation, medially with or without short angular protrusion); horns complanate, inner edge (axial view) without accessory lobe(s) or denticle(s). Dorsal eye parts distinctly elliptic (width varying according to species, eyes separated by 3–5 times their widths). Clypeofrontal ridge variably distinct (straight or arcuate, angularly connected to clypeogenal suture or ridge). Clypeal apex more or less reflexed, broadly bisinuate to truncate-rounded. Genal border rounded, clypeogenal transition at most very slightly indented or outwardly angular at border. Basic colour black or (rufous) brown, non-patterned, with or without metallic lustre (but lacking shifting light reflection of satin elytra under different angles of view). Pronotum anteromedially gibbous (at most shortly, bluntly bifid), or more or less broadly depressed; if depressed, surface, at most, posteriorly delimited by blunt protrusion(s) (never with set of sharp forward projections, denticles, or tubercles). Anterolateral section of pronotal edge straight or concave, angle distinct. Parameres with slender, moderately curved, usually tapering tip. Body length usually 8–12 mm	*Onthophagus catenatus* group, 2
–	Combination of characters different	other groups of *Onthophagus*
2	Pronotum largely rugulate-punctate, its broadly rounded anterior gibbosity with scale-like rugulation, anterolaterally with long setae. Lateral border of pronotum distinctly, abruptly angulate (in dorsal view). Ridge connecting base of vertexal horns medially topped with forward angle. Generally black, lacking metallic lustre. Clypeofrontal ridge strongly pronounced, distinctly, evenly arcuate. Males and females similar. Body length over 10 mm	*Onthophagus kokosquamatus*
–	Pronotum more finely punctate, not rugulate, glabrous. Lateral border of pronotum simply rounded at the middle. Frontal disc on either side delimited by ridge extending along gena and eye	3
3	Vertexal horns at base with intervening angle or more or less isolated conical protrusion. Pronotum with distinct pair of blunt forward protrusions (rim bisinuate in dorsal view) behind depressed anterior declivity	*Onthophagus kokodanus* (2 subspecies), 4
–	Vertexal horns at base simply connected, intervening elevation not angular. Pronotum with anterior declivity simply gibbous (at most with very slight midline impression), or broadly deplanate with transversely arcuate, blunt, posterior ridge	5
4	Vertexal median protrusion shifted forward, in major males more or less isolated, conical; base of horns dilated (plate-like) up to about half of horn length, tip of horns laterally beyond eyes. Elytra distinctly matt, strial punctures all equally fine	*Onthophagus kokodanus kokodanus*
–	Vertexal median protrusion angulate, situated on basal intervening connection of horns; base of horns broad, gradually narrowing to tapering tip, which does (in axial view) laterally extend over (not beyond) eyes. Elytra shining, strial punctures generally more strongly impressed	*Onthophagus kokodanus hagenaltus*
5	Anteromedian pronotal declivity gibbous, convex from disc down, surface behind horns shallowly concave. Punctures of elytral striae from base to apex increasingly strong, more broadly impressed and affecting adjacent interstriae. Smaller, body length 8–10 mm	*Onthophagus catenatus*
–	Anterior declivity of pronotum broadly deplanate, sloping down from generally arcuate, transverse posterior rim. Punctures of elytral striae over entire length all equally impressed. Larger, body length ca 11.5 mm	*Onthophagus abmisibilus*

## Descriptions

### Onthophagus abmisibilus sp. n.

urn:lsid:zoobank.org:act:F6BBF729-53C1-4DEA-AA33-7912A142A44A

http://species-id.net/wiki/Onthophagus_abmisibilus

**Figures 1–8.**
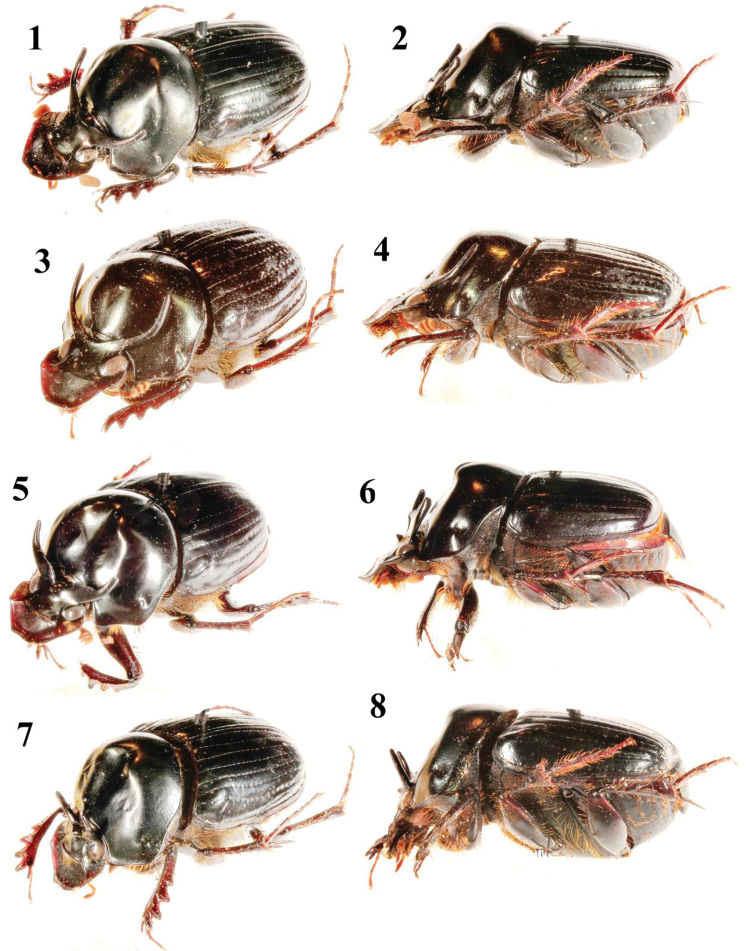
Habitus (oblique and lateral views) of *Onthophagus* males (holotypes, except **3–4**). **1–2** *Onthophagus abmisibilus* **3–4** *Onthophagus catenatus*, vicinity of Mt Hagen **5–6** *Onthophagus kokodanus kokodanus* **7–8** *Onthophagus kokodanus hagenaltus*.

Figs 1–2 12, 17 22–26

#### Type-material.

**Holotype,** male: (RMNH), Indonesia, : West New Guinea, Papua, Star Mountains, Abmisibil, 1900–2200 m, 4°38'S, 140°33'E, 29.i–09.ii.2005, T. Lackner. Locality description with landscape pictures: http://www.papua-insects.nl/about%20Papua/Starmountains/Star%20Mountains.htm (accessed 30.iii.2012).

#### Diagnosis.

Male of this species has two very distinctive features: the concave (in axial view), non-angulate crest of the plate connecting the bases of the vertexal horns, and the rather flat, faintly metallic anterior declivity of the pronotum. Dorsum black-brown, largely matt (particularly elytra). Elytral strial punctures all distinct, fine, equal-sized, and only slightly crenulating interstriae. Lateral border of pronotum distinctly rounded at some distance from anterolateral angle. Pronotum with widely bisinuate, blunt crest behind anterior declivity. Clypeofrontal ridge low, almost straight, on either end angularly connected at clypeogenal ridge. Most of dorsal side finely to minutely, sparsely punctate. Clypeal tip broadly, slightly reflexed, apical border bisinuate. Dorsal eye parts broadly elliptic, separated by slightly over three eye-widths. Pygidium abundantly, finely, distinctly punctate. Body length ca 11.5 mm, *Onthophagus catenatus* being smaller, 8–10 mm.

#### Description

(holotype, male). Body length ca 11.5 mm. Habitus generally convex, robust. Colour of dorsal side black, generally matt (microreticulate), with slight metallic lustre; ventral parts largely black, partly matt (microreticulate); legs black-brown, shiny. Dorsal side and pygidium virtually glabrous (apart from any inconspicuous micro-stubbles); ventral side and legs with numerous long, light-brown setae.

Head shiny, sparsely micropunctate throughout. Clypeal border broadly, distinctly marginate, sides very slightly rounded (almost straight) from genae to apex, the latter bisinuate, slightly reflexed, shortly lobiform; clypeogenal transition at border very obtusely angular, on either side with almost straight ridge extending past end of very weakly curved, distinct clypeofrontal ridge onto frontal side. Genal border evenly, widely rounded. Vertex between posterior end of eyes with pair of long, basally broad horns, connected at base by transverse, slightly concave, laterally sinuate (plate-like) elevation (in axial view); horns widely, evenly arcuate upward, directed laterad, over and beyond eyes (in axial view), evenly tapering to blunt tip; distal section of horn rounded on all sides. Frontal disc limited by fine frontogenal ridge, on either side extending along eye. Dorsal eye parts widely elliptic, with ca 28 facet rows across widest point. Ratio of interocular distance to maximum (transverse) eye width ca 3.3.

Pronotum generally strongly convex, surface matt, with cupreous lustre; disc posteriorly slightly convex (midline impression almost effaced), anterior declivity broadly deplanate, posterior rim slightly transversely arcuate-sinuate, blunt; anterior and lateral borders of pronotum marginate; anterior section of lateral border slightly concave; anterolateral angle rectangular, shortly rounded; posterior section of lateral border slightly sinuate, posterolateral angle rounded; base finely marginate, with very obtuse basomedian angle. Most of pronotal disc and medial surface in general sparsely micropunctate; lateral declivities with larger, but fine, more abundant punctation (surface microreticulate).

Elytra generally black, virtually matt (microreticulate); scutellum indistinct in dorsal view, shape of base and apex unmodified; anterior half of epipleuron distinctly punctate-setose. Elytra broad, with 8 straight to slightly curved, fine, distinct striae; stria 7 slightly sinuate in front, extending onto shining humeral umbone; striae fine, punctures fine but distinct, widely separated (mostly 4–8 puncture diameters), slightly crenulating interstrial edges. Interstrial surfaces virtually flat to slightly convex (from disc to lateral declivity of elytra), with sparse, inconspicuous micropunctation.

Antennal club medium-brown, scapus unmodified. Mentum shallowly emarginate in front. Prothoracic sides with coxal-marginal ridge distinct, curving forward onto lateral border, much of surface annulate-punctate-setose, including large, seta-bearing hemi-punctures. Anterior lobe of metasternum slightly convex, abundantly punctate-setose, disc micropunctate; metasternal sides and adjacent parts matt, abundantly to densely annulate-punctate-setose. Abdominal ventrites matt, each laterally with row of seta-bearing annulate punctures. Pygidium black, matt (microreticulate), with abundant, shallow, fine punctation, without macro-setae; surface slightly convex, base with transverse ridge, apex marginate.

Legs robust. Protibia with 3+1 larger external denticles (distal 3 longer, sub-acuminate), separated by some fine serration; proximal serration consisting of 8 smaller denticles; apico-external denticle oblique to tibial axis; apical edge slightly round, with strong, down-curved, elongate-acuminate spur; protibial underside with low longitudinal crest; protarsus unmodified. Profemur robust, underside finely punctate and with numerous larger seta-bearing hemi-punctures. Meso- and metafemoral undersides also finely punctate, with fewer large seta-bearing hemi-punctures. Meso- and metatibiae strongly dilated distad to transversely subelliptic crest, which is fringed with fine fossorial spines; external side with ca 4 sets of distinct, spine-bearing fossorial protrusions. Tarsi generally slender, with well-developed sickle-shaped claws; meso- and metatarsomeres 1 long, straight, unmodified. Spurs on meso- and metatibiae elongate-acuminate. Relative length proportions of metatibial spur to metatarsomeres 1–5: ca 28, 33, 13, 9, 7, 14.

Parameres narrow (dorsal view), tapering, curved downward (lateral view), Fig. 17.

Measurements (in dorsal view): maximum width of head 3.4 mm, median length of pronotum 3.5 mm, maximum width of pronotum 6.0 mm, sutural length of elytra 5.5 mm, maximum width of combined elytra 6.3 mm.

#### Variability and sexual dimorphism.

Only male holotype is known.

#### Etymology.

The name of the new species is derived from the nameof the type locality, a village in the central mountain chain, on the Indonesian side of the Indonesian-Papuan New Guinea border; to be treated as masculine noun.

### Onthophagus catenatus

Lansberge, 1883

http://species-id.net/wiki/Onthophagus_catenatus

Figs 3–4 13, 18 27–32

#### Material examined.

104 males and females, 11 collection records. Papua New Guinea: 18 mi N Port Moresby, Brown River, 14–15/vii/1974, Peck, 10ft, forest: dung, 32 spm., in CMNC. 30 mi N Port Moresby, Brown River, 15–16/vi/1974, Peck, 3 spm., in CMNC. Kokoda Trail, 20 mi E Port Moresby, 16–18/vii/1974, Peck, 10ft, forest: dung, 35 spm., in CMNC. 15–16/vii/1974, Peck, 10ft, forest: dung, 15 spm., in CMNC. 16–18/vii/1974, Peck, 10ft, forest: dung, 2 spm., in CMNC. Kokoda Trail, 34 mi E Port Moresby, 17–18/vii/1974, Peck, 2200ft, 3 spm., in CMNC. 16–17/vii/1974, Peck, 2200ft, 9 spm., in CMNC. 17–18/vii/1974, Peck, 2200ft, 1 spm., in CMNC. Kuk Exp. Station nr Mt Hagen, 04–12/vii/1974, Howden, 1 spm., in CMNC (pictured, body length ca 9 mm). Madang: Baitabag, 6^o^08'19"S – 145^o^46'34"E, 30/i/2000, Anderson, 100m, rainforest litter, 2 spm., in CMNC. [Indonesia:] “N. Guinea \ Amberbaki”, 1 male (syntype?). Additional material seen from both Papua New Guinea and West New Guinea localities, including the Aru Islands.

#### Diagnosis.

The following combination of three features should distinguish *Onthophagus catenatus* from its close relatives, at least the major males: evenly concave (in axial view), non-angulate crest of the plate connecting the bases of the male vertexal horns; generally convex (gibbous), relatively smooth anterior declivity of the pronotum; and particularly the posteriorly increasing size of the strial punctures of the elytra – normally both males and females should be recognizable from this punctation. Dorsum brown to black (occasionally bicolorous, elytra may be light-brown), shining, usually with distinct metallic lustre (particularly on pronotum). Lateral border of pronotum distinctly rounded (not angular) at some distance from anterolateral angle. Clypeofrontal ridge very low, virtually straight, on either end angularly connected at clypeogenal ridge. Most of dorsal side finely to minutely, sparsely punctate. Clypeal tip slightly reflexed, apical border rounded to truncate. Dorsal eye parts broadly elliptic, separated by 3–3.5 eye widths. Pygidium abundantly, finely, distinctly punctate. Females with transverse vertexal ridge. Body length usually 8–10 mm.

#### Comment.

A male in the Gillet drawers of the IRSNB, agreeing with van Lansberge’s (1883) description, bears type label and originates from the region mentioned in the original description (northern New Guinea) – it may be one of the syntypes.

### Onthophagus kokodanus sp. n.

urn:lsid:zoobank.org:act:F55E685F-8F8A-43A0-A3DF-CEE39BA5A3C0

http://species-id.net/wiki/Onthophagus_kokodanus

#### Comments.

Under this species name two subspecies are characterized, one from the Kokoda Trail region E of Port Moresby, at about 700 m, and one from the Mt Hagen region at about 1800 m. They differ in structural development and in microsculpture, as diagnosed hereafter and in the key above.

### Onthophagus kokodanus kokodanus subsp. n.

http://species-id.net/wiki/Onthophagus_kokodanus_kokodanus

Figs 5–6 14, 19 33–38

#### Type-material.

41 males and females, 4 collection records. **Holotype** male (CMNC) from Papua New Guinea: 34 mi E Port Moresby, Kokoda Trail, 2000 ft, 17–18.vii.1974, S. Peck, T43–44.

**Paratypes:** Papua New Guinea: Kokoda Trail, 34 mi E Port Moresby, 17–18/vii/1974, Peck, 2000ft, 6 spm., incl. holotype, in CMNC. Kokoda Trail, 30–34 mi E Port Moresby, 16–17/vii/1974, Peck, 2200ft, 4 spm., in CMNC. Kokoda Trail, 34 mi E Port Moresby, 17–18/vii/1974, Peck, 2200ft, 17 spm., in CMNC. 16–17/vii/1974, Peck, 2200ft, 14 spm., in CMNC.

#### Diagnosis.

The pair of interconnected, basally broad (in axial view), plate-like vertexal horns in the major male, with the median conical tubercle just in front, constitutes the primary feature of this subspecies – at least in major males. Lateral border of pronotum distinctly rounded at about 0.4 of length behind anterolateral angle (not angular, in dorsal view, Fig. 34). Pronotum with blunt bisinuate rim behind broadly depressed, more or less concave anterior declivity, all this reduced in minors. Clypeofrontal ridge low, slightly arcuate, ends on either side angularly connected at clypeogenal ridge. Most of dorsal side finely to minutely, sparsely, evenly punctate. Clypeal tip distinctly, broadly reflexed, apical border bisinuate. Dorsal eye parts broadly elliptic, separated by slightly over three eyewidths. Elytra sericeous, virtually matt, sparsely micropunctate; strial punctures very fine, widely separated, all similar, not stronger caudad. Pygidium sparsely to abundantly, very finely punctate. Colour generally black-brown, moderately shiny, without metallic lustre. Body length usually 10–12 mm.

The presence of an isolated conical protrusion right in front of the horns is shared with other New Guinea species, like *Onthophagus heurni* Gillet, 1930 and *joliveti* Paulian, 1973, which may be confusing. The males of both these species, however, have on their horns, which are more or less erect, a distinct basal-internal lobe or denticle, and their eyes are narrow.

#### Description

(holotype, male). Body length ca 12 mm. Habitus generally convex, robust. Colour of dorsal side (brown-)black, generally moderately shiny, elytra sericeous; ventral parts largely black, matt (microreticulate); legs dark-brown, shiny. Dorsal side and pygidium virtually glabrous (apart from any inconspicuous micro-stubbles); ventral side and legs with numerous long, light-brown setae.

Clypeal border broadly, distinctly marginate, sides virtually straight from genae to bisinuate, reflexed, shortly lobiform apex; clypeal surface shiny, with sparse, minute punctation; clypeogenal transition at border obtusely angular (anterolateral corner of gena slightly elevated), on either side with straight ridge to slightly arcuate, distinct clypeofrontal ridge. Genal and frontal surface minutely punctate as on clypeus; genal border evenly, widely rounded. Vertex between posterior end of eyes with pair of long, complanate, basally very broad horns, connected at base, with distinct conical tubercle medially on anterior side; horns strongly directed laterad (beyond eyes, in axial view), evenly curving upward to tapering, blunt tip, inner edge of horns sinuate (in axial view); horn surface also minutely, sparsely punctate. Frontal disc limited by fine frontogenal ridge, on either side extending along eye. Dorsal eye parts widely elliptic, with ca 28 facet rows across widest point. Ratio of interocular distance to maximum (transverse) eye width ca 3.2.

Pronotum generally strongly convex, surface shiny; disc posteriorly slightly convex (midline impression virtually effaced), anterior surface broadly depressed, medially shallowly concave up to posterior, strongly bisinuate, transverse protrusion coming from disc forward; anterior and lateral borders of pronotum marginate; anterior section of lateral border slightly concave; anterolateral angle rectangular, shortly rounded; posterior section of lateral border slightly sinuate, posterolateral angle rounded; base medially finely marginate along very obtuse basomedian angle. Most of pronotal surface finely, sparsely punctate, anterior depression minutely, sparsely punctate, this punctation interspersed with vague micropunctation.

Elytra generally weakly shiny to matt (sericeous); scutellum indistinct in dorsal view, shape of base and apex unmodified; anterior half of epipleuron distinctly punctate-setose. Elytra broad, with 8 straight to slightly curved, fine, distinct striae; stria 7 distinctly sinuate in front, extending onto shining humeral umbone; strial punctures very fine, widely separated (ca 10 puncture diameters), hardly crenulating interstrial edges. Interstrial surfaces virtually flat to very slightly convex (from disc to lateral declivity of elytra), with sparse, inconspicuous micropunctation.

Antennal club light-brown, scapus unmodified. Mentum shallowly emarginate in front. Prothoracic sides with coxal-marginal ridge distinct, curving forward onto lateral border, much of surface annulate-punctate-setose, including large, seta-bearing hemi-punctures. Anterior lobe of metasternum slightly convex, abundantly punctate-setose, disc micropunctate; metasternal sides and adjacent parts matt, abundantly to densely annulate-punctate-setose. Abdominal ventrites sericeous, each laterally with strip of seta-bearing annulate punctures. Pygidium black, weakly shiny (sericeous), with sparse to abundant, shallow, very fine punctation, without macro-setae; surface slightly convex, base with transverse ridge, apex marginate.

Legs very robust. Protibia with 3+1 larger external denticles (distal 3 longer, sub-acuminate), separated by some serration; proximal serration consisting of 5–6 smaller denticles; apico-external denticle oblique to tibial axis; apical edge slightly rounded, with strong, acuminate, down-curved spur; protibial underside with low longitudinal crest; protarsus unmodified. Profemur robust, underside finely punctate, and with numerous larger seta-bearing hemi-punctures. Meso- and metafemoral undersides also finely punctate, with fewer large seta-bearing hemi-punctures. Meso- and metatibiae strongly dilated distad to transversely subelliptic crest, which is fringed with fine fossorial spines and longer setae; external side with ca 4 sets of distinct, spine-bearing fossorial protrusions. Tarsi generally slender, with well-developed sickle-shaped claws; meso- and metatarsomeres 1 straight, unmodified. Spurs on meso- and metatibiae elongate-acuminate. Relative length proportions of metatibial spur to metatarsomeres 1–5: ca 14, 17, 6, 4, 3, 6.

Parameres narrow (dorsal view), tapering, curved downward (lateral view), Fig. 19.

Measurements in dorsal view: maximum width of head 3.7 mm, median length of pronotum 4.4 mm, maximum width 6.4 mm, sutural length of elytra 4.8 mm, maximum width of combined elytra 6.7 mm.

#### Variability and sexual dimorphism.

Strongly varying in the development of the vertexal armature. Females have a complex transverse vertexal ridge (Fig. 38), not the variably long horns like in males (note the minor male in Fig. 15, and beware of extremely minor males looking like females, as in the next subspecies). Female vertexal ridge with sides laterally obtusely angulate, thence sloping to eye, medially with robust knob; pronotum with pair of blunt, forward protrusions topping anterior declivity. Body length 9–12 mm.

#### Etymology.

Name refers to the Kokoda Trail, the type locality; to be treated as masculine noun.

### Onthophagus kokodanus hagenaltus subsp. n.

http://species-id.net/wiki/Onthophagus_kokodanus_hagenaltus

Figs 7–8 16, 20 39–44

#### Type material.

5 males, 2 collection records. **Holotype** male (CMNC) from Papua New Guinea: 25 mi NE Mount Hagen, 6000 ft, 5.vii.1974, H.F. Howden.

**Paratypes:** Papua New Guinea: 25 mi NE Mount Hagen, 05/vii/1974, Howden, 6000ft, 3 spm., incl. holotype, in CMNC. Western Highlands: Mt. Hagen, 05–08/vii/1974, Peck, 6000ft, oak forest, 2 spm., in CMNC

#### Diagnosis.

The vertexal horns of this subspecies are more upright, even in major males, with angular tubercle on top of the interconnecting basal ridge. Dorsum black-brown, largely shining (particularly the elytra), with slight greenish metallic lustre. Elytral strial punctures all equally small, distinct, scatteredly crenulating finely punctate interstriae. Lateral border of pronotum distinctly rounded at about 0.4 of length behind anterolateral angle (not angular, in dorsal view, Fig. 40). Pronotum usually with blunt bisinuate rim behind depressed, medially more or less concave anterior declivity. Clypeofrontal ridge low, very slightly arcuate, ends on either side angularly connected to clypeogenal ridge. Most of dorsal side finely, sparsely punctate. Clypeal tip distinctly, broadly reflexed, apical border bisinuate. Dorsal eye parts broadly elliptic, separated by about three eye widths. Pygidium abundantly, finely, distinctly punctate. Body length usually 10–11 mm. Female unknown.

Apparently a close highland relative of the preceding subspecies.

#### Description

(holotype, male). Body length ca 11 mm. Habitus generally convex, robust. Colour of dorsal side black, generally shiny, with slight metallic lustre; ventral parts largely black, partly matt (microreticulate); legs black-brown, shiny. Dorsal side and pygidium virtually glabrous (apart from any inconspicuous micro-stubbles); ventral side and legs with numerous long, light-brown setae.

Clypeal border broadly, distinctly marginate, sides virtually straight from genae to bisinuate, reflexed, shortly lobiform apex; clypeal surface shiny, with sparse to abundant, minute punctation; clypeogenal transition at border obtusely angular, slightly elevated, on either side with straight ridge curving to end of virtually straight, distinct clypeofrontal ridge. Genal and frontal surface sparsely, minutely punctate; genal border evenly, widely rounded. Vertex between posterior end of eyes with pair of long, basally very broad horns, connected at base by angular ridge, with distinct anteromedian angle; horns slightly curved, directed upright behind eyes (not beyond, in axial view), evenly tapering to blunt tip, inner edge of horns evenly concave (in axial view); horn surface with convex anterior surface, minutely, sparsely punctate. Frontal disc limited by fine frontogenal ridge, on either side extending along eye. Dorsal eye parts widely elliptic, with ca 28 facet rows across widest point. Ratio of interocular distance to maximum (transverse) eye width ca 3.0.

Pronotum generally strongly convex, surface shining, with greenish lustre; disc posteriorly slightly convex (midline impression effaced), anterior surface broadly depressed, medially shallowly concave, up to posterior, strongly bisinuate, transverse protrusion coming from disc forward; anterior and lateral borders of pronotum marginate; anterior section of lateral border slightly concave; anterolateral angle rectangular, shortly rounded; posterior section of lateral border slightly sinuate, posterolateral angle rounded; base finely marginate, with very obtuse basomedian angle. Most of pronotal surface finely, sparsely punctate, anterior depression minutely, sparsely punctate, this punctation interspersed with vague micropunctation; lateral declivities more matt (microreticulate).

Elytra generally shiny, with greenish metallic lustre; scutellum indistinct in dorsal view, shape of base and apex unmodified; anterior half of epipleuron distinctly punctate-setose. Elytra broad, with 8 straight to slightly curved, fine, distinct striae; stria 7 slightly sinuate in front, extending onto shining humeral umbone; strial punctures distinct, small, widely separated (mostly 5–8 puncture diameters), distinctly crenulating interstrial edges. Interstrial surfaces virtually flat to very slightly convex (from disc to lateral declivity), with sparse to abundant, fine micropunctation.

Antennal club medium-brown, scapus unmodified. Mentum shallowly emarginate in front. Prothoracic sides with coxal-marginal ridge distinct, curving forward onto lateral border, much of surface annulate-punctate-setose, including large, seta-bearing hemi-punctures. Anterior lobe of metasternum slightly convex, abundantly punctate-setose, disc micropunctate; metasternal sides and adjacent parts matt, abundantly to densely annulate-punctate-setose. Abdominal ventrites matt, each laterally with row of seta-bearing annulate punctures. Pygidium black, weakly shiny (sericeous), with abundant, shallow, fine punctation, without macro-setae; surface slightly convex, base with transverse ridge, apex marginate.

Legs robust. Protibia with 3+1 larger external denticles (distal 3 longer, sub-acuminate), separated by some serration; proximal serration consisting of 7 smaller denticles; apico-external denticle oblique to tibial axis; apical edge slightly rounded, with down-curved spur (tip worn away); protibial underside with low longitudinal crest; protarsus unmodified. Profemur robust, underside finely punctate, and with numerous larger seta-bearing hemi-punctures. Meso- and metafemoral undersides also finely punctate, with fewer large seta-bearing hemi-punctures. Meso- and metatibiae strongly dilated distad to transversely subelliptic crest, which is fringed with fine fossorial spines; external side with ca 4 sets of distinct, spine-bearing fossorial protrusions. Tarsi generally slender, with well-developed sickle-shaped claws; meso- and metatarsomeres 1 straight, unmodified. Spurs on meso- and metatibiae elongate-acuminate. Relative length proportionsof metatibial spur to metatarsomeres 1–5: ca 12, 13, 6, 4, 3, 6.

Parameres narrow (dorsal view), tapering, curved downward (lateral view), Fig. 20.

Measurements in dorsal view: maximum width of head 3.2 mm, median length of pronotum 3.5 mm, maximum width 5.5 mm, sutural length of elytra 4.6 mm, maximum width of combined elytra 5.8 mm.

#### Variability and sexual dimorphism.

Female unknown; females probably have a transverse vertexal ridge, not the variably long horns of the males. Strongly varying: there is a minor male looking like a female, having a transverse vertexal ridge (Fig. 44), its aedeagus being similar to that of the holotype. Body length 9.5–11 mm.

#### Etymology.

Name refers to the highland nature of the type locality.

### Onthophagus kokosquamatus sp. n.

urn:lsid:zoobank.org:act:DEDE0A16-B300-4FB8-AC2C-D36556913EB2

http://species-id.net/wiki/Onthophagus_kokosquamatus

Figs 9–11, 21 45–49

#### Type material.

15 males and females, 4 collection records. **Holotype** male (CMNC) from Papua New Guinea: 34 mi E Port Moresby, Kokoda Trail, 2000 ft, 16–17.vii.1974, S. Peck, T43–45.

**Paratypes:** Papua New Guinea: Kokoda Trail, 30–34 mi E Port Moresby, 16–17/vii/1974, Peck, 2200ft, 2 spm., in CMNC. Kokoda Trail, 34 mi E Port Moresby, 16–17/vii/1974, Peck, 2200ft, 4 spm., incl. holotype, in CMNC. 17–18/vii/1974, Peck, 2200ft, 4 spm., in CMNC. Kokoda Trail, Kauai River, Manari, 12–14/viii/1976, Kukal, 700m, rain forest: dung, 5 spm., in CMNC.

#### Diagnosis.

The strongly rugulate-punctate pronotum, with the peculiar squamiform texture on the anterior gibbosity, together with its large size, should distinguish *Onthophagus kokosquamatus* from its relatives, or for that matter, any known Papuasian congeners. Bases of both vertexal horns interconnected by robust ridge topped with distinct forward angle. Lateral margin of pronotum distinctly angular at about 0.4 of length behind anterolateral angle (Fig. 46). Pronotal surface behind horns with numerous long setae. Distinctly arcuate clypeofrontal ridge well developed, its ends on either side angularly connected at clypeogenal ridge. Clypeogenal border obtusely angular (full-face view). Clypeal tip distinctly, broadly reflexed, apical crest bisinuate. Dorsal eye parts elliptic, less broad than in the preceding species, separated by about 4.5 eye widths. Most of head surface abundantly, finely punctate; female clypeus transversely rugulate. Elytral interstriae on disc sparsely, indistinctly punctate, lateral interstriae more densely punctate; strial punctures very fine, widely separated, all similar. Pygidium very densely, distinctly punctate. Metasternum superficially prow-shaped in front, behind transverse rim. Colour generally black-brown, without any metallic lustre (elytra lacking reflections shifting in relation to angle of view). Male and female very similar. Body length usually 11–12 mm. For smaller potential relatives, cf. comments below.

#### Description

(holotype, male). Body length ca 11.5 mm. Habitus convex, robust. Colour of dorsal side black, generally moderately shiny, forebody heavily (rugulate-)punctate; ventral parts largely black, partly shiny, strongly punctate; legs brown-black, shiny. Dorsal side and pygidium locally with some longer light brown setae (apart from inconspicuous micro-setae); ventral side and legs with numerous long, light-brown setae.

Clypeal border broadly, distinctly marginate, sides virtually straight from genae to apex, the latter bisinuate, reflexed, shortly lobiform; clypeal surface shiny, with abundant, fine punctation; clypeogenal transition at border obtusely angular, on either side with straight ridge to arcuate, very distinct clypeofrontal ridge. Genal surface finely, abundantly punctate as on clypeus, surface, like sides of clypeus, superficially transverse rugulate; genal border evenly, widely rounded. Vertex between posterior end of eyes with pair of long horns, which are convex in front, basally broad, and are connected at base by high, medially angulate ridge (axial view); horns strongly directed laterad (over eyes), evenly curving upward to tapering (on posterior side thickened) tip, their surface abundantly, very finely punctate. Frontal disc finely, abundantly punctate between slight genal sutures. Dorsal eye parts broadly elliptic, with 13–15 facet rows across widest point. Ratio of interocular distance to maximum (transverse) eye width ca 4.5.

Pronotum generally strongly convex; disc slightly convex (midline impression virtually effaced by heavy punctation); anteromedian surface strongly, evenly bulbous from disc forward; sides, behind horns of head, distinctly concave (with numerous long, decumbent setae), sloping down to anterolateral corner; anterior and lateral borders of pronotum marginate; anterior section posteriorly, at ca 0.4 of total length, with distinct angle, border very slightly concave; anterolateral angle rectangular, rounded; posterior section of lateral border strongly sinuate, posterolateral angle distinct; base medially slightly marginate along very obtuse basomedian angle. Most of pronotal surface densely rugulate-punctate, rugulation on anteromedian bulb squamiform (like reptile skin); anterolateral concavities largely smooth, many punctures on anterolateral surface looking like small horseshoes.

Elytra generally weakly shiny, disc almost matt; scutellum indistinct in dorsal view, shape of base and apex unmodified; epipleuron distinctly punctate-setose. Elytra broad, with 8 straight to slightly curved, fine, distinct striae; stria 7 distinctly sinuate in front, extending onto punctate, shining humeral umbone; strial punctures very fine, widely separated (ca 10 puncture diameters), hardly crenulating interstrial edges. Interstrial surfaces flat to very slightly convex (from disc to lateral declivity), with inconspicuous fine punctation, gradually larger, denser, more distinct, slightly rugulate to lateral interstriae.

Antennal club light-brown, scapus unmodified. Mentum shallowly emarginate in front. Prothoracic sides with coxal-marginal ridge distinct, curving forward onto lateral border, most of surface densely annulate-punctate-setose. Anterior lobe of metasternum slightly convex (very slight median ridge in front), abundantly hemi-punctate-setose; disc abundantly, finely punctate; metasternal sides and adjacent parts matt, densely to crowdedly annulate-punctate-setose. Abdominal ventrites matt, laterally all crowdedly annulate-punctate-setose. Pygidium black, distal part shiny, with dense, distinct, simple punctation, with very few longer macro-setae; surface slightly convex, base with transverse ridge, apex marginate.

Legs very robust. Protibia with 3+1 larger external denticles (distal 3 longer, sub-acuminate), separated by some serration; proximal serration consisting of 5–6 smaller denticles; apico-external denticle oblique to tibial axis; apical edge protruding, rounded (upper side view), with strong, acuminate, down-curved spur; protibial underside with low longitudinal crest; protarsus unmodified. Profemur robust, underside finely punctate, and with numerous larger seta-bearing hemi-punctures. Meso- and metafemoral undersides also finely punctate, with fewer large seta-bearing hemi-punctures. Meso- and metatibiae strongly dilated distad to transversely sinuate-subelliptic crest, which is fringed with fine fossorial spines and setae; external side with ca 4 sets of distinct, spine-bearing fossorial protrusions. Tarsi generally slender, with well-developed sickle-shaped claws; meso- and metatarsomeres 1 straight, unmodified. Spurs on meso- and metatibiae elongate-acuminate. Relative length proportions of metatibial spur to metatarsomeres 1–5: ca 15, 14, 6, 4, 3, 4.

Parameres narrow, with narrow spatuliform tip (dorsal view), tapering, curved downward (lateral view), Fig. 21.

Measurements in dorsal view: maximum width of head 3.5 mm, median length of pronotum 4.5 mm, maximum width 6.2 mm, sutural length of elytra 4.9 mm, maximum width of combined elytra 6.5 mm.

#### Variability and sexual dimorphism.

General body shape of both sexes is very similar. Female clypeus transversely rugulate throughout. Small morphs have less developed horns and obsolescent intervening median angle. Body length 10.5–12 mm.

#### Comments.

Some smaller *Onthophagus* (up to ca 8 mm long) with narrow dorsal eye parts and satin-iridescent elytra, from various parts of New Guinea (including the Bismarck Islands), are superficially similar to *Onthophagus kokosquamatus*, for instance by their completely rugulate-punctate pronotum and pair of curved vertexal horns (in both sexes). These include *Onthophagus irianus* Balthasar, 1969 and *novaeirlandiae* Balthasar, 1969 (cf. his key couplet 78/79). By the shifting light reflections from their elytral disc, absent in *kokosquamatus*, they are reminiscent of species in the Papuasian group of species around *Onthophagus iris* Sharp, 1875. A study of this group is under way, and in that context the position of these smaller *kokosquamatus*-like forms and other potential relatives will certainly be reconsidered.

#### Etymology.

Specific epithet was derivedfrom geographic origin of the new species and peculiar squamiform pronotal sculpture.

**Figures 9–21.**
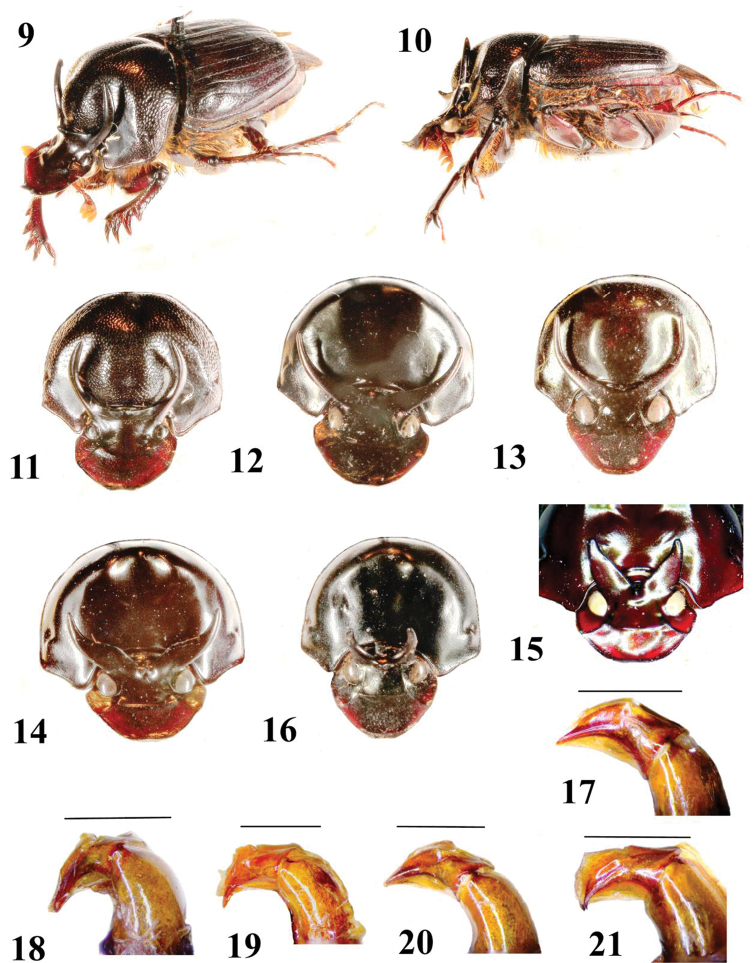
*Onthophagus* males (holotypes, except **13, 15, 18**) . **9–11,** **21** *Onthophagus kokosquamatus*; **12, 17** *Onthophagus abmisibilus*; **13, 18** *Onthophagus catenatus*, vicinity of Mt Hagen **14, 15, 19** *Onthophagus kokodanus kokodanus*, **15** minor male E Pt Moresby **16, 20** *Onthophagus k hagenaltus*. **9–10** habitus (oblique and lateral views) **11–16** head and pronotum in dorsofrontal view **17–21** parameres in lateral view (scales 1 mm).

**Figures 22–32.**
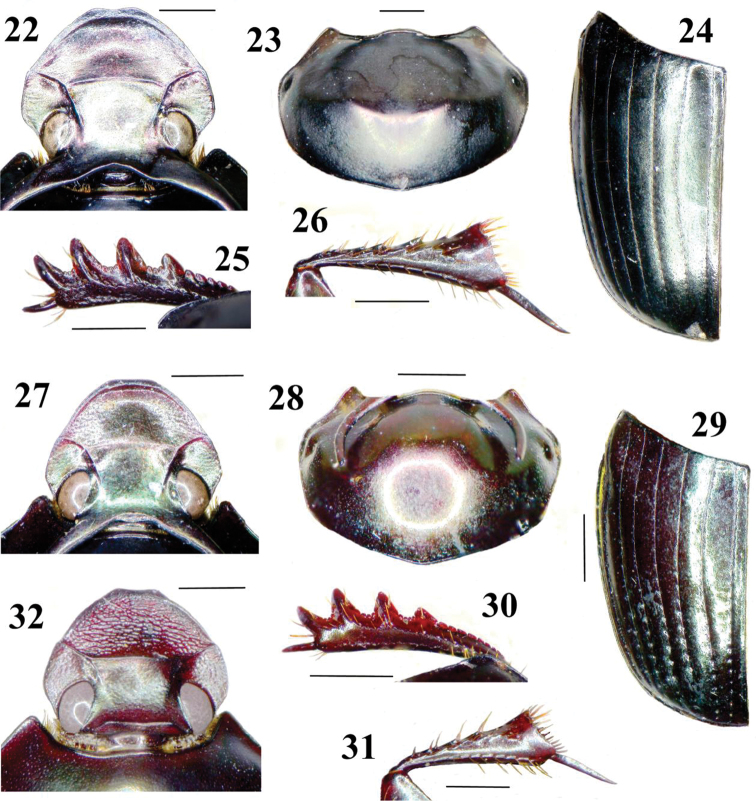
Details of morphology of *Onthophagus*. **22–26** *Onthophagus abmisibilus*, holotype **27–32** *Onthophagus catenatus*, male, vicinity of Mt Hagen **32** female, N Pt Moresby **22, 27, 32** head in dorsal view **23, 28** pronotum in dorsal view **24, 29** elytron in dorsal view **25, 30** protibia, upper side **26, 31** metatibia, underside. Scales 1 mm.

**Figures 33–44.**
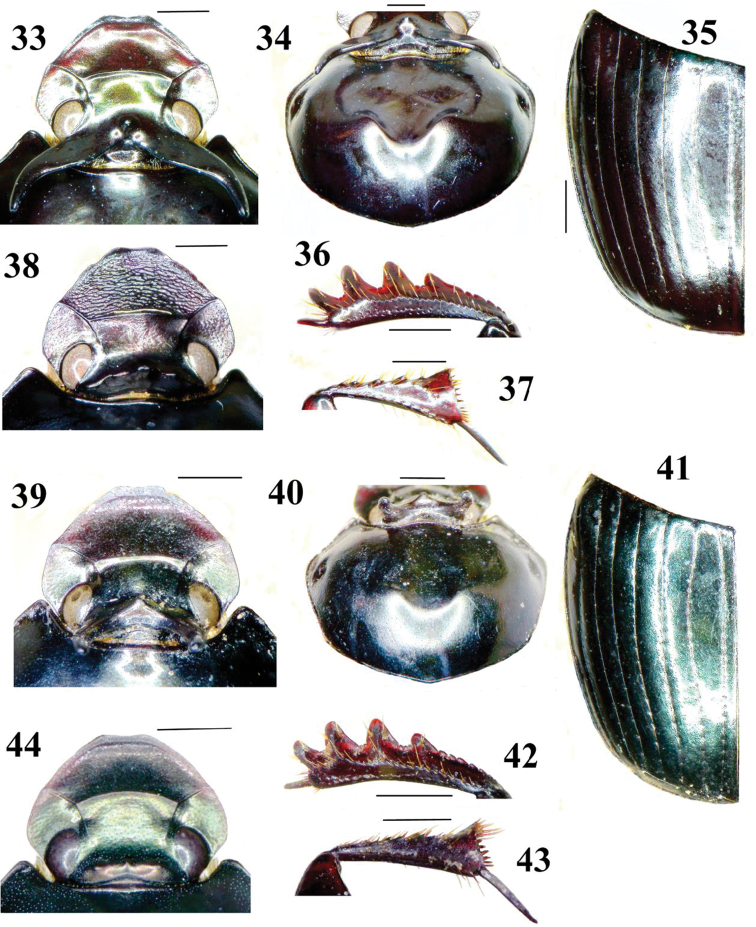
Details of morphology of *Onthophagus* (holotypes, except **38, 44**). **33–38** O. *k. kokodanus*, 38 female paratype, E Pt Moresby **39–44** *Onthophagus kokodanus hagenaltus* **44** minor male paratype, Mt Hagen **33, 38, 39, 44** head in dorsal view **34, 40** pronotum and head armature in dorsal view **35, 41** elytron in dorsal view **36, 42** protibia, upper side **37, 43** metatibia, underside. Scales 1 mm.

**Figures 45–49.**
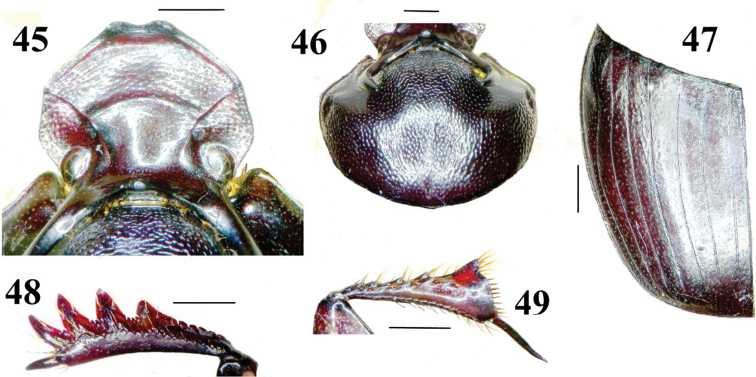
Details of morphology of *Onthophagus kokosquamatus* (male holotype) **45** head in dorsal view **46** pronotum and head armature in dorsal view **47** elytron in dorsal view **48** protibia, upper side **49** metatibia, underside. Scales 1 mm.

## Supplementary Material

XML Treatment for Onthophagus abmisibilus

XML Treatment for Onthophagus catenatus

XML Treatment for Onthophagus kokodanus

XML Treatment for Onthophagus kokodanus kokodanus

XML Treatment for Onthophagus kokodanus hagenaltus

XML Treatment for Onthophagus kokosquamatus

## References

[B1] Balthasar V (1969) Neue *Onthophagus*-Arten von Neu-Guinea und den benachbarten Inseln. Acta Entomologica Musei Nationalis Pragae 38: 361-408

[B2] Gillet JJE (1930) Onthophagides de Nouvelle Guinée. Nova Guinea (Zoologie) 15 (1929): 411-434

[B3] Krikken J Huijbregts J (2012) Taxonomy of Papuasian *Onthophagus*: twenty new species and their relatives (Coleoptera: Scarabaeidae: Scarabaeinae). Tijdschrift voor Entomologie 155: 73–131

[B4] Krikken J Huijbregts J (in press) New Guinea *Onthophagus*: taxonomy of ten new small, unicolour species (Coleoptera: Scarabaeidae: Scarabaeinae). Zootaxa. 10.11646/zootaxa.3619.5.1 26131489

[B5] van Lansberge JW (1883) Révision des *Onthophagus* de l’archipel Indo-Néerlandais, avec description des espèces nouvelles. Notes from the Leyden Museum 5: 41-82

[B6] Paulian R (1973) Récoltes de M.P. Jolivet en Nouvelle-Guinée. Coléoptères Scarabaeidae Onthophaginae. Bulletin de la Société Entomologique de France 77(1972): 215–217 [Publication date p. 320]

